# STM patterned nanowire measurements using photolithographically defined implants in Si(100)

**DOI:** 10.1038/s41598-018-20042-8

**Published:** 2018-01-29

**Authors:** A. N. Ramanayaka, Hyun-Soo Kim, Ke Tang, X. Wang, R. M. Silver, M. D. Stewart, J. M. Pomeroy

**Affiliations:** 1 National Institute of Standards & Technology, Gaithersburg, Maryland 20899 USA; 2 Department of Electrical and Computer Engineering, University of Maryland, College Park, Maryland 20742 USA; 3 Department of Materials Science and Engineering, University of Maryland, College Park, Maryland 20742 USA; 4 Chemical Physics Program, University of Maryland, College Park, Maryland 20742 USA

## Abstract

Using photolithographically defined implant wires for electrical connections, we demonstrate measurement of a scanning tunneling microscope (STM) patterned nanoscale electronic device on Si(100). By eliminating onerous alignment and complex lithography techniques, this approach is accessible to researchers in smaller efforts who may not have access to tools like electron beam lithography. Electrical contact to the nanodevices is achieved by implanting patterned, degenerately doped wires in the substrate using photolithography and commercial low energy ion implantation. We bring several isolated, implanted wires to within the STM scanner’s field of view where the STM can detect and smoothly draw contiguous patterns that directly overlap with implant lines for electrical connections. This overlapping provides a two-dimensional (2D) overlap interface with the 2D electron system, in contrast to many state-of-the-art methods that rely on contacting an exposed edge. After the STM pattern is phosphine dosed and overgrown with silicon, photolithography is then used again to align (≈ 160 *μ*m)^2^ aluminum contact pads onto (≈ 200 *μ*m)^2^ implanted areas at the ends of the wires. We present detailed results that optimize the spacing of neighboring wires while maintaining electrical isolation after heating to > 1200 °C, a step required for *in situ* Si surface preparation.

## Introduction

Electron spins confined to shallow donors in isotopically enriched ^28^Si are promising candidates for qubits due to their scalability and extremely long coherence times^1,2^. Fabrication of these nanoscale devices, unfortunately, is challenging and generally requires massive research efforts to overcome major difficulties in areas such as lithography, interface control, and external contacts. None-the-less, the use of ultra high vacuum (UHV) STM (scanning tunneling microscope) lithography techniques^3,4^ for patterning planar nanoscale electronic devices has enabled fabrication and measurement of atom scale wires to, e.g., verify the persistence of Ohm’s law at the atomic limit^5^, fabricate atomically precise tunnel junctions^6^ for single electron detection, and demonstrate a donor based triple quantum dot device demonstrating serial electron transport through three quantum dots^7^. Unfortunately, interfacing and fabricating external electrical contacts to these nanoscale devices continues to be unreliable and requires complex, time consuming, highly specialized nanofabrication techniques and tools. Even with the most advanced, state-of-the-art fabrication tools, simply finding the buried nanodevice to contact is often unsuccessful, and every device requires a customized pattern for electrical contacts^8^.

Here we demonstrate a standardized photolithography scheme to simplify interconnecting a nanoscale device to external electrical contacts. The electrical connection from the nanoscale device to the external contacts is achieved by P ion implanting photolithographically defined areas to create degenerately doped regions in Si substrates (see Methods for further information). Typically, Si substrates are prepared for STM by flash annealing to > 1200 °C for approximately a minute total, which can cause a significant amount of implant diffusion. The implant diffusion constrains the minimum spacing of isolated, neighboring implanted wires, yet at least 4 contact wires need to be available for measuring devices, preferably within a single STM scan frame. The use of preimplanted electrical connections to measure STM lithographic patterns was previously reported for probing nanowire transport^9^, however there the substrates were not prepared by high temperature flashing of the Si samples. We define the following as a set of specifications needed in order to ensure electrical isolation, maximize the number of electrical contacts and minimize STM frame size: (a) at least 4 implant wires must be available for device operation; (b) all of the implant wires must be within a single STM frame (10 *μ*m × 10 *μ*m); (c) the implant wires must survive high temperature processing at 1200 °C for at least 2 minutes with a ${R}_{\square } < 1\,k{\rm{\Omega }}$ (resistance per square) at 4 K; (d) all the wires must remain electrically isolated from each other (*R* ≥ 10 GΩ at 4 K, i.e., limited by the current capabilities of this measurement system); and, (e) STM identification and alignment to the preimplanted contacts must be straightforward, e.g., require < 1 hour.

**Figure 1:**
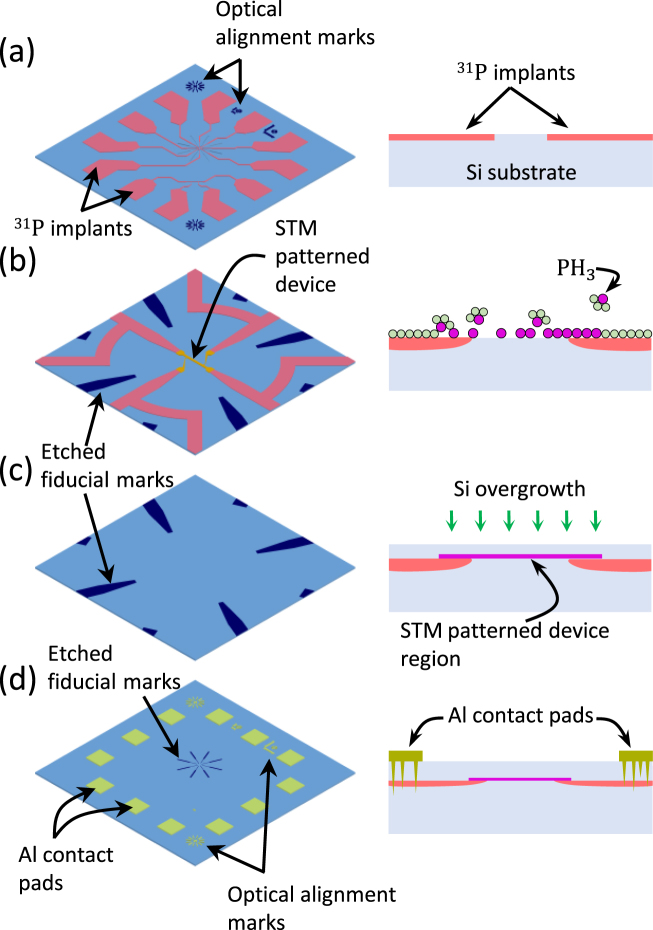
A schematic representation of the implant based contact process for STM patterned devices. For each step shown, the left is a pseudo-plan view and the right is a cross-section of the step. First (**a**), the heavily doped contact wires are defined by using photolithography and low energy ion implantation. Secondly, after the wafer is etched with STM alignment marks and diced, implanted chips are loaded into a UHV system for high temperature processing, H-passivation and STM lithography. The etched fiducial marks, see (**b**) and (**c**), guide the STM during its coarse positioning to locate the preimplanted wires. Upon completion of STM lithography (**b**), the pattern is dosed with phosphine and heated to incorporate the dopants. Note that the overlap between the STM pattern and the implant wire define the interface between the implant wire and the 2D electron gas. Then, (**c**) a capping layer of Si is deposited to encapsulate the device. Finally, the sample is removed from the chamber and (**d**) Al metal contacts are deposited and patterned by photolithography.

An overview of our design and processing strategy that satisfies these specifications is shown in Fig. 1 (further details of the materials and methods are included in the Methods section). A plan view and cross section of the full die ready to be inserted into UHV is shown in Fig. 1(a). Photolithography has been used to define alignment marks and then macroscopic implant regions (pink) around the perimeter, which are later used for metal contacts, and fine wires that radially reach into the center frame to where the STM patterning will be performed. Additionally, shallow (≈ 100 nm) etch features are aligned to the implant lines to provide visible markers for coarse alignment of the STM (discussed further later). A zoom in of the center region is shown in Fig. 1(b), where four implant lines (pink) are shown as well as the ends of four shallow etch lines (blue). The next steps occur within the UHV system; the sample is flashed to > 1200 °C to remove the oxide, form a 2 × 1 reconstruction and facilitate hydrogen passivation. The hydrogen passivation is then selectively removed by the STM to create a pattern of reactive silicon that will form the device, e.g., a wire connecting two leads with additional wires for four point probing, shown in yellow. After the hydrogen is patterned, the sample is exposed to phosphine gas [Fig. 1(b) - right] and thermally activated to transfer the hydrogen pattern to heavily doped P regions in the silicon. The fine area is shown again in Fig. 1(c), where the patterned device and implant lines are overgrown with silicon while still in UHV, encapsulating the device and the implant lines. Finally, the full die is shown again in Fig. 1(d), where photolithography is used to pattern macroscopic aluminum contact pads over the large implant regions (≈ 200 *μ*m)^2^ and and the alignment of the metal photo mask to the sample is realized by using the etched optical alignment marks. Finally, a low-temperature thermal spiking establishes electrical contact through the silicon capping layer.

This strategy dramatically simplifies the contemporary strategies for aligning and contacting STM patterned nanodevices. Specifically, no sample specific patterning or alignment is required outside of the STM patterning step, in contrast to typical contacting schemes. Our strategy does not use any electron beam lithography, at any stage. Furthermore, the bulk of the *ex situ* (non-UHV) processing is done on the wafer scale, i.e., the alignment marks, implant and shallow etch features are all performed at the wafer level. Since a typical 100 mm wafer can yield ≈ 100 of the 4 mm × 10 mm chips used in the vacuum processing, the productivity benefit of the wafer scale processing can be enormous after only a few chips. Finally, since this approach provide overlap between two conducting planes, rather than drilling holes or slicing faces through the delta layer plane, the quality and reliability of the contacts may be better once it is optimized. But, in order to realize this approach, detailed knowledge of the dopant diffusion for the complex thermal histories used in the UHV sample preparation is needed.

## Results

Another unstated requirement in developing this preimplant strategy is to minimize the disruption of the successful processes being carried out during the UHV stages of the nanodevice fabrication. Paramount amongst these is surface preparation of the Si(100) templates used for STM-based patterning of the nanodevices. This typically begins with 8 hours degassing at ≈ 600 °C followed by staged ramping and several “flashes” (several second excursions) where the samples reach temperatures ≥ 1200 °C (see UHV processing in methods section). At temperatures above 1000 °C, P diffuses significantly in Si which can lead to unintended electrical leakage or shorting. Therefore, detailed knowledge of P diffusion specific to these samples and conditions is needed. Modeling of the post-processing distribution of P atoms is not straightforward due to the proximity of the surface and the complicated nature of the temperature profile, which is not well represented by a singular time and temperature typically used to evolve a dopant profile (see UHV processing in methods section). Furthermore, as these are shallow implants in Si, one must account for bulk and surface diffusion and different surface configurations, e.g. dimers, step edges, etc^10,11^ to calculate the P diffusion accurately. Attempts to numerically estimate diffusion lengths and the onset of shorting are insufficient to provide the confidence for designing a robust solution. Therefore, to avoid complicated analysis and building in too many assumptions, we first designed an experiment to determine acceptable spacing between neighboring implant lines for different high temperature processing protocols that ensures electrical isolation. Further discussion will be provided later on our paramterization of diffusion and comparisons to diffusion estimates shown in Table 1.

**Table 1:** For each sample, the integrated thermal activation paramter ${{D}}_{{\rm{\Sigma }}}^{\ast }$ is shown in the second column, followed by the aggregate time spent at each 1000 °C, 1100 °C, and 1200 °C. Using the ${D}_{{\rm{\Sigma }}}^{\ast }$ parameter for each sample, we calculate equivalent activation times (*t*_*eq*_) if the sample had sat at a single temperature of either 1100 °C or 1200 °C. The next two columns provide single dopant bulk and surface diffusion lengths using *E*_*a*_ = 3.5 eV^12^ for 3D, *E*_*a*_ = 0.94 eV^11^ for 2D and *D*_0_ = 3.85 cm^2^/s^19^ for both bulk and surface diffusion. Finally, the equivalent time at 1100 °C is used to estimate the spreading of the implanted dopants “edge” using an online diffusion calculator, corresponding to a dopant density of 3.8 × 10^18^ cm^3^.

Sample	${{\boldsymbol{D}}}_{{\boldsymbol{\Sigma }}}^{{\boldsymbol{\ast }}}$	Total time (s)^a^			*t*_*eq*_ (min)^b^		Diffusion length (*μ*m)^b^		*l*_*c*_ (*μ*m)^c^
		1000 °C	1100 °C	1200 °C	1100 °C	1200 °C	*l*_3*D*_	*l*_2*D*_	
D1	0.084 ± 0.003	60	0	0	0.10	0.01	0.02	2.09 × 10^3^	—
D2	2.5 ± 0.3	55	48	23	2.81	0.38	0.10	3.99 × 10^3^	0.23
D3	5.1 ± 0.4	69	54	45	5.86	0.79	0.14	5.73 × 10^3^	0.34
D4	6.1 ± 0.4	0	97	51	6.97	0.94	0.15	5.78 × 10^3^	0.37
D5	7.5 ± 0.6	0	7	60	8.56	1.15	0.17	4.53 × 10^3^	0.43
D6	9.1 ± 0.5	0	151	93	10.34	1.39	0.19	7.04 × 10^3^	0.46
D7	26.2 ± 0.5	52	1816	23	29.95	4.03	0.32	16.08 × 10^3^	0.78

^a^The total times reported for each sample for each temperature is the time that the sample reached 95% of the corresponding temperature and we estimate the uncertainty to be  ± 2 s. ^b^Relative uncertainty for equivalent anneal time (*t*_*eq*_) and the diffusion lengths (*l*_3*D*_, and *l*_2*D*_) are estimated as < 10%. ^c^*l*_*c*_ is the broadening of the critical concentration for metal-insulator transition relative to as implanted dopant profile due to annealing at 1100 °C for *t* = *t*_*eq*_ time using publicly accessible dopant profile calculator^20^.

### Determining the minimum separation between implant lines

**Figure 2:**
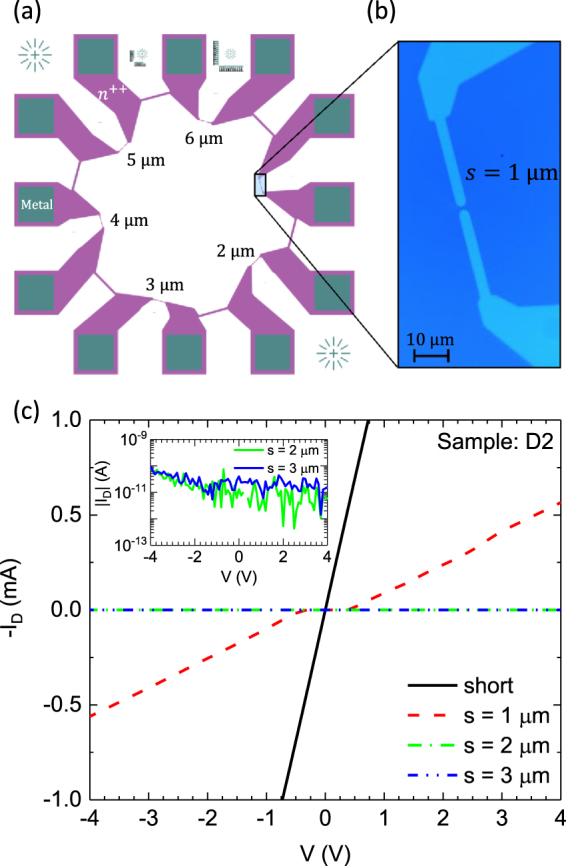
To determine sufficient implant spacing for different processing conditions, a test structure with several gap spacings per die was used - plan view in (**a**). On each die are multiple devices, each with two implanted leads separated by a distance *s* (here *s* varies from 1 *μ*m to 6 *μ*m), each with a short to adjacent lead to allow four point measurements. (**b**) An optical microscope image of a device with *s* = 1 *μ*m gap is shown. (**c**) Representative I-V characteristics from devices on die 2 after high temperature flash annealing are shown. The shorted (control) wire clearly shows a Ohmic behavior. The device with *s* = 1 *μ*m spacing also shows a considerable leakage current (*I*_*D*_) and is shorted for practical purposes. The devices with *s* = 2 *μ*m and *s* = 3 *μ*m spacing have no appreciable leakage current (|*I*_*D*_| < 100 pA) through the device (see insert).

To be used as either gates or Ohmics in silicon based nanodevices, we define “electrically isolated” to be ≥ 10 GΩ at 4 K, i.e., 100 pA of current leakage at 1 V. As shown in Fig. 2(a), we designed test structures with 16 devices of different gap spacings in order to determine which gaps maintain ≥ 10 GΩ isolation after high temperature processing (as measured to nearest neighbors). Each device consists of two implant lines 3 *μ*m wide separated by a gap (*s*) such that 1 *μ*m ≤ *s* ≤ 6*μ*m. The example test structure shown in Fig. 2(a) contains devices with 1 *μ*m ≤ *s* ≤ 6*μ*m gaps. The optical micrograph in Fig. 2(b) shows a device with *s* = 1 *μ*m separation prior to thermal processing (the features are not optically visible once activated). Each lead on a given device is connected to its own Ohmic contact pad and the one of its neighboring device. This allows us to maximize the number of devices per die while still enabling four terminal resistance measurements on each device. Additionally, good electrical connection between the wire bonds, metal contact pads and implanted silicon can be verified at low temperatures independently from the gap resistances. In addition to these 16 “gap” devices, the set of test devices also includes a shorted (*s* = 0) implant line (3 *μ*m wide and 54 *μ*m long) to determine the implant line resistance after the high temperature processing.

For each of the gap spacings, current vs. voltage (IV) measurements are made at < 4 K after high temperature processing. Example four-point IV measurements of a shorted (*s* = 0) implant line (black) and gap devices with *s* = 1 *μ*m, *s* = 2 *μ*m and *s* = 3 *μ*m are shown in Fig. 2(c). The IV for the shorted implant line is linear (Ohmic) with a resistance of approximately 730 Ω. Considering the number of squares, ${R}_{\square }\approx 41\,{\rm{\Omega }}$ and satifies specification (c). For the device with *s* = 1 *μ*m, the IV is also linear (except for |*V*_*s*_| < 0.7 V) with a relatively low resistance, indicating that thermally activated diffusion shorted the gap. The other two devices shown in Fig. 2(c) with *s* = 2 *μ*m and *s* = 3 *μ*m remain isolated with <100 pA at |*V*_*s*_| ≤ 4 V of leakage current. The measured leakage current for these two devices is shown in Fig. 2(c) insert, which is near the floor of our measurement system. Therefore, for this particular thermal process, gap spacings ≥ 2 *μ*m remain electrically isolated and meet specification (d). However, a range of thermal processing steps is often used and we need to generalize this result to determine the sensitivity of the electrical isolation to the thermal history.

**Figure 3:**
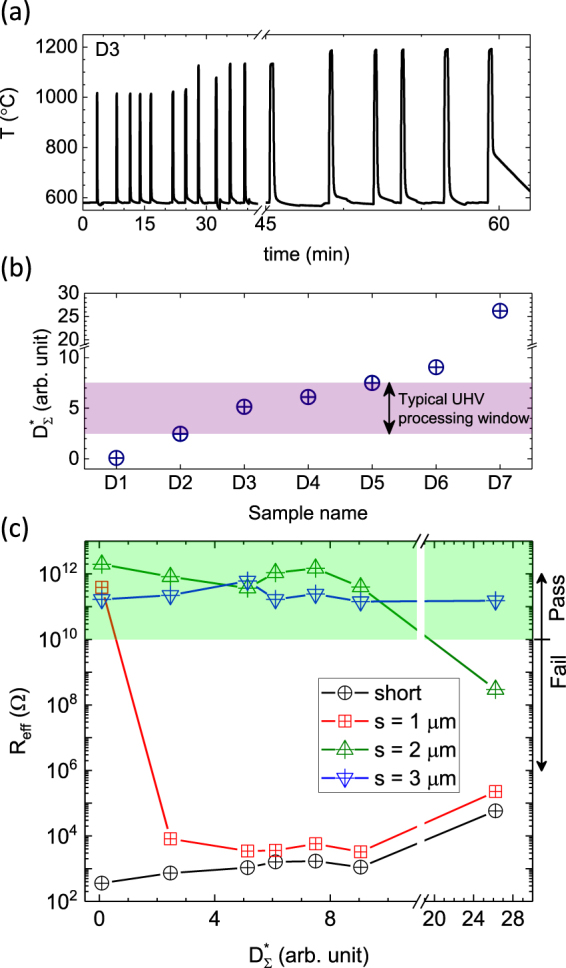
As shown in (**a**), the thermal history of a sample subjected to a typical UHV flash anneal process is complex, with extended periods at ≈ 600 °C and brief intervals > 1200 °C. (**b**) To account for this history and compare samples, a Boltzmann weighted time integral (see text) is used to calculate the total effective thermal activation (${D}_{{\rm{\Sigma }}}^{\ast }$) for the different thermal processes tested. The ${D}_{{\rm{\Sigma }}}^{\ast }$ values span a wide range in this experiment, going beyond the shaded area representing ${D}_{{\rm{\Sigma }}}^{\ast }$ typical for UHV flash anneals. Derived relative uncertainty for ${D}_{{\rm{\Sigma }}}^{\ast }$ is ≤ 10% and is due to the uncertainty in the temperature measurements. Each point on the x-axis represents a different sample, as labeled. (**c**) The effective four terminal resistances (*R*_*eff*_) for a shorted implant line and test devices with *s* = 1 *μ*m, 2 *μ*m, 3 *μ*m are shown for each of the high temperature flash anneal shown in (**b**). All the device with 2 *μ*m separation show ≥ 100 GΩ isolation at temperature < 4 K except for *D*7 where ${D}_{{\rm{\Sigma }}}^{\ast }\approx 26$. All devices with ≥ 3 *μ*m separation show ≥ 100 GΩ isolation for all thermal activations shown in (**b**). The shaded area in (**c**) represents the resistances that meet the specifications, i.e., *R*_*eff*_  ≥ 10 GΩ. Calculated relative uncertainty for *R*_*eff*_ is ≤ 0.1% and is dominated by the error in voltage measurements and leakage current of the measurement setup.

As mentioned earlier, the complexity and variability of the in-vacuum thermal processing from run to run complicates quantitative comparison between samples, which is important for determining how sensitive the selected spacing is to the details of a particular thermal process. An example temperature vs. time profile for an *in situ* sample preparation is shown in Fig. 3(a) (after the completion of an overnight degassing procedure at 600 °C). In this case, the sample was flashed several times to ≈ 1000 °C while ensuring that the chamber pressure remained < 1.3 × 10^−7^ Pa (< 1 × 10^−9^ Torr), then several times to ≈ 1100 °C, before the final flashes at ≈ 1200 °C. Table 1 summarizes the approximate aggregate time spent by each sample at *T* = 1000 °C, *T* = 1100 °C and *T* = 1200 °C. This history can be used to estimate diffusivity $D={D}_{0}{e}^{-\frac{{E}_{a}}{{k}_{B}T}}$^12^, given a particular choice of *E*_*a*_, the activation energy, and *k*_*B*_, the Boltzmann constant. In all likelihood, the diffusion of P atoms can be influenced by both bulk and surface diffusion mechanisms. Furthermore, extended thermal treatments can cause sublimation of phosphorus that depletes the surface dopant density^13^ and reduces the efficacy of contacts. The shallow nature of the implant opens up the possibility of many possible diffusion or depletion mechanisms. For reference, we have calculated bulk 3D diffusion lengths, surface diffusion lengths and expected dopant profile broadening (see Table 1) and none of these match with the experimental findings, discussed further below. Therefore, to parameterize the thermal history of each sample and allow comparison with equivalent single temperature processes, we define the integrated effective thermal activation of implanted ions as:

1 $${D}_{{\rm{\Sigma }}}^{\ast }=\frac{k}{2}\,\sum _{i}({e}^{-\frac{{E}_{a}}{{k}_{B}T({t}_{i+1})}}+{e}^{-\frac{{E}_{a}}{{k}_{B}T({t}_{i})}})\,({t}_{i+1}-{t}_{i})$$

where *k* is a scaling parameter that equals to 10^11^ added for convenience and has no physical significance, and *T*(*t*) represents the temperature profile of the UHV flash anneal process, specific to the sample being considered. For the parameterization, we use *E*_*a*_ = 3.5 eV^12^ to provide an approximate thermal weighting. The ${D}_{{\rm{\Sigma }}}^{\ast }$ values calculated on several chips are shown in Fig. 3(b) (and Table 1), where the shaded range spanning ${D}_{{\rm{\Sigma }}}^{\ast }\approx 5$ represents the range typically used for preparing chips in UHV. Thermal activation of the dopants for this set was accomplished in multiple ways: sample D1 was heated to 1000 °C in a rapid thermal annealer (RTA) for one minute; samples D2, D3 and D5 were UHV flash annealed using our standard flash anneal protocol (the variation in ${D}_{{\rm{\Sigma }}}^{\ast }$ is primarily due to different times spent at 1200 °C); samples D4 and D6 were repeatedly flash annealed two and three times, respectively; and, finally, sample D7 was flash annealed once followed by a 30 min anneal at 1100 °C.

For each of the different ${D}_{{\rm{\Sigma }}}^{\ast }$ values, the effective resistance, *R*_*eff*_, of the gaps were calculated by taking the mean of the resistance measured from 0.5 V to 4 V at temperature < 4 K after the thermal processing. These *R*_*eff*_ values are shown in Fig. 3(c) for the shorted implant line (*s* = 0) and the devices with *s* = 1 *μ*m, *s* = 2 *μ*m and *s* = 3 *μ*m gaps. The green shaded region indicates values that satisfy specification (d). For sample D1, which has the lowest ${D}_{{\rm{\Sigma }}}^{\ast }$, all three devices remain electrically isolated with *R* > 10 GΩ. For the samples D2 through D6 (with ${D}_{{\rm{\Sigma }}}^{\ast }$ value ranging from 3 to 10), the devices with *s* = 1 *μ*m gaps are insufficiently isolated, while the devices with a lead separation of *s* = 2 *μ*m and *s* = 3 *μ*m gaps still have > 10 GΩ isolation. For the sample D7 with ${D}_{{\rm{\Sigma }}}^{\ast }\approx 26$, both *s* = 1 *μ*m and *s* = 2 *μ*m gaps are shorted and only the device with *s* = 3 *μ*m gap remains isolated.

To help provide a bit more intuitive sense of the thermal parameter ${D}_{{\rm{\Sigma }}}^{\ast }$, the approximate time spent at each high temperature processing step for each sample is shown in Table 1. Then, using the known ${D}_{{\rm{\Sigma }}}^{\ast }$ values calculated for each sample, we can ask what time is equivalent (*t*_*eq*_), if the sample had sat at a single temperature? These values are shown in the 6th and 7th column for *T* = 1100 °C and *T* = 1200 °C and are useful for comparison with other common industrial activation or annealing processes. Using the same formulation as Eq. 1 to sum over the thermal history, we can also calculate a predicted single atom diffusion length for bulk and surface diffusion as well. But single particle diffusion lengths tell us very little about where the boundary of a degenerately doped implant region will move during a thermal process and the values found bear no resemblance to the data in Fig. 3(c). To better estimate the degenerate dopant boundary motion, we use the calculated *t*_*eq*_ for *T* = 1100 °C and the initially implanted dopant profile in a publicly accessible dopant profile diffusion calculator. We estimate the spreading of critical concentration for metal-insulator transition relative to that of as implanted dopant profile (see *l*_*c*_ in the final column of Table 1). Even though the estimated broadening is approximately 2.4 times larger than the diffusion length from bulk diffusion (*l*_3*D*_ of Table 1), i.e. *l*_*c*_/*l*_3*D*_ ≈ 2.4, the estimated spreading still falls short of the experimental findings. As a test for the use of ${D}_{{\rm{\Sigma }}}^{\ast }$ as a parameter, we performed the same calculations using *t*_*eq*_ for *T* = 1000 °C and *T* = 1200 °C (not shown) and found the predicted spreading was within 10% of the *T* = 1100 °C case. That finding supports ${D}_{{\rm{\Sigma }}}^{\ast }$ as good proxy for predicting the strength of a given thermal process.

The thermally activated diffusion impacts not only the fidelity of the isolation, but also the ${R}_{\square }$ of the implants (specification c) at low temperature as the dopants spread out. Examining the resistance of the shorts on samples D1 up to D6 [see Fig. 3(b)], the values increase a factor of 3 over approximately two orders of magnitude increase in ${D}_{{\rm{\Sigma }}}^{\ast }$. For sample D7, however, the resistance jumps (> 50×) compared to that of a sample went through a typical UHV flash anneal, e.g., sample D3, corresponding to an ${R}_{\square }\approx 3.2\,{\rm{k}}{\rm{\Omega }}$, failing specification (c). Using the *t*_*eq*_ at *T* = 1100 °C to estimate the dopant spreading, we see that for D7 the peak dopant density has fallen from the initial 5 × 10^20^ cm^3^ to ≈ 1 × 10^20^ cm^3^. Consequently, the standard UHV flash anneal (${D}_{{\rm{\Sigma }}}^{\ast }\mathrm{ < 10}$) doesn’t seem to affect the resistance of the implant lines significantly, indicating that the highly doped electrical wires withstand the high temperature processing of Si substrates, except at very high ${D}_{{\rm{\Sigma }}}^{\ast }$.

Since all the gap devices *s* ≥ 2 *μ*m (30 devices in total) remain electrically isolated for the practical range of ${D}_{{\rm{\Sigma }}}^{\ast }$, we can use that design rule to construct a pattern that satisfies specifications (a) through (d) listed above and expect > 10 GΩ when using ${D}_{{\rm{\Sigma }}}^{\ast }\mathrm{ < 10}$. For example, considering that a circle circumscribed within a 10 *μ*m × 10 *μ*m square has a perimeter of ≈ 31 *μ*m, we can bring 10 implant lines that are each 1 *μ*m wide while still ensuring ≥ 2 *μ*m spacing between the lines. Using the full perimeter of the square allows 12 wires, etc. This leaves specification (e), identification and alignment within the STM, which we demonstrate next.

### STM patterned nanowire device

**Figure 4:**
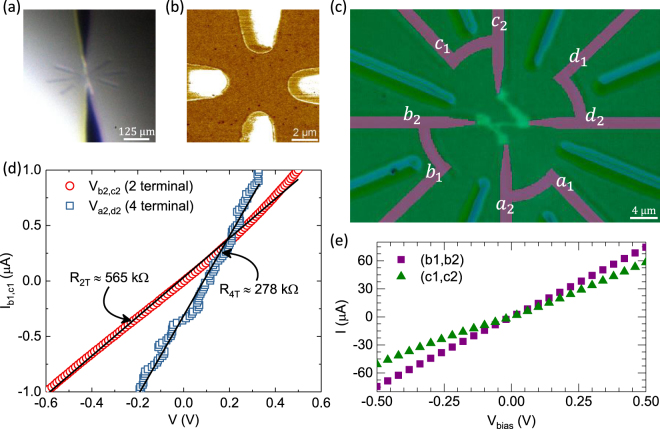
Optical image (**a**) taken through a vacuum window of the STM tip (bottom) and its reflection (top) visually aligned to the etched alignments marks. (**b**) AC phase contrast of an STM image (DC: *V*_*bias*_ = −2 V, *I*_*set*_ = 100 pA, AC: *f* = 80 kHz, *V*_*rms*_ = 200 mV) of the substrate near implant lines clearly shows the location of the implant wire, to which the write pattern can be aligned. (**c**) A differential interference contrast (Nomarski) microscopy image of the finished sample with STM patterned device is shown. The dark pink color traces are drawn in to indicate the location of the P implant regions, and the lighter color traces are the fiducial markers for coarse positioning the STM. Light green colored region in the center is the STM patterned, P doped wire connecting the leads *a*_2_, *b*_2_, *d*_2_, and *c*_2_, respectively, allowing a four point measurement of the segment from *a*_2_ to *d*_2_. (**d**) 2, and 4 terminal voltages measured across (*b*_2_, *c*_2_) and (*a*_2_, *d*_2_) versus the current bias applied across (*b*_1_, *c*_1_). (**e**) shows the I-V characteristics of the preimplant lines (*b*_1_, *b*_2_) and (*c*_1_, *c*_2_).

Utilizing the results in the previous section, we have designed a four wire implant pattern to demonstrate identification and electrical contacting to an STM patterned 2D nanodevice. As shown in the annotated optical micrographs in Fig. 4(c), we have designed an implant pattern based on eight radial wires (dark pink) in which every other wire is cross-linked to its adjacent wire (e.g., *b*_1_ connected to *b*_2_), improving the measurements by eliminating parasitic resistances in the connecting circuits (leads, wire bonds, etc.). Four of the implant lines continue toward the center for direct connection to the STM written pattern, terminating when the tips are on a 8 *μ*m diameter circle to leave the clear space in which the device is patterned. Between each of the implant lines is a shallow radial etch feature (light blue) so that the implant lines and the etch features share a common center point and the radial etch features are terminated when the tips are on a 26 *μ*m diameter circle to leave the clear space for the STM navigation. These etch features are visible through the coarse alignment telescope used to orient the STM tip with the sample, as shown in Fig. 4(a), where the tip can be seen entering from the bottom of the image and its reflection from surface goes out the top. Using the telescope in combination with the etch features, our first tip approach is within ≈ 5 *μ*m of the implant/etch center point. At a pre-anneal depth of 100 nm, these features are deep enough to be seen by the optical telescope, yet shallow enough to scan across with the STM when using a large tunneling gap. Since each of the etch features points toward the center, the proximity of the initial approach combined with clever choices of scan size and rates allow one to center the frame on the pattern within about 30 minutes. The implanted lines themselves also have some residual topography that persists after the thermal processing that can be used to aid alignment. Consequently, considering all features point to the center, identification of correction vectors is straightforward.

Since the phosphorus is implanted into a lightly boron doped substrate, the spectroscopic capabilities of the STM can also be used to provide a direct image of the electrically active region of the fine area. We have implemented this by mixing an AC modulation of the gap voltage (at a frequency above the feedback bandwidth) onto the DC component and then performing a lock-in measurement on the feedback current. This allows the local density of states (LDOS) around the DC bias point to be imaged in real time (at the same scan rate as the imaging). As shown in Fig. 4(b), we find excellent contrast between the P implanted regions and the surrounding substrate. This imaging method substantially reduces the risk of misalignment between the STM and implant patterns. The combination of appropriate optical telescope selection, the radial etch features and the STM imaging satisfy the final specification (e) that we initially determined.

Finally, we use the implant templates and specifications described in this paper to demonstrate successful electrical connection and measurement of an STM patterned nano-wire. After thermal preparation of a preimplanted Si(100) substrate, the chip was hydrogen terminated and moved to the STM. After centering the STM frame on the implant pattern, the hydrogen was selectively depassivated to draw large overlay regions (≈ 1 *μ*m × 1 *μ*m) onto the ends of the implant lines and narrow (≈ 300 nm) wires that connect the implant lines *b*_2_ ↔ *d*_2_, *c*_2_ ↔ *d*_2_ and *b*_2_ ↔ *a*_2_. The electrical contact depends only on the overlay and the size of the nanodevice does not depend on the overlay regions. As a proxy for any selected nanodevice, we have used a  ≈ 300 nm wire. After STM patterning, the features were dosed with phosphine gas to selectively dope the patterned regions with P (several depassivation and dosing cycles were used in the course of the full pattern). Then a brief heating cycle to activate the P was used before overgrowing the patterned region with epitaxial silicon (additional details are provided in the Methods section). The actual nanowire pattern can be seen in the optical micrograph shown in Fig. 4(c). After removal from the vacuum chamber, aluminum contact pads are patterned in a single, chip level photolithography step that defines an etch mask. After patterning, a brief thermal cycle ensures good electrical contact between the aluminum and the large implant region beneath it [e.g., the spikes seen in Fig. 1(d)-right].

Once complete, the sample was mounted on a closed-cycle cryocooler measurement system that cooled the sample to < 4 K. At this temperature all of the aluminum to P doped contacts had low (< 30 kΩ) resistances as determined by pairwise measurements, e.g., *a*_1_ ↔ *a*_2_, etc. Example IV measurements of these implant line pairs are shown in Fig. 4(e). For the electrical connections between the nanowire and the implants, we found that the resistances for two (*b*_2_ and *c*_2_) of the four contacts were good and two (*a*_2_ and *d*_2_) of them were poor (insufficient for current, but sufficient for voltage probes). Using the good contacts to source and drain current through the STM written nanowire, we first performed a “two terminal” (2 pt) measurement by sending current from *b*_1_ ↔ *c*_1_ and measuring the voltage drop from *b*_2_ ↔ *c*_2_, shown in Fig. 4(d) in red. Note that while this is a “two-terminal” measurement of the wire since it includes the nanowire to implant contact resistance, the independent voltage probes (*b*_2_ and *c*_2_) still eliminate the parasitic resistance from all the external connections and implant wires, etc. These measurements were performed in a current bias configuration from ± 1 *μ*A, spanning a voltage range of more than a volt while remaining essentially linear.

Next we changed to a “four terminal” (4 pt) configuration by using *a*_2_ and *d*_2_ as voltage probes to measure a subsection of the wire between *b*_2_ and *d*_2_, thus eliminating the voltage drop across the nanowire contacts. The four-terminal IV measurement is shown in blue, and is also linear over the entire range of the measurement, with a resistance diminished roughly in proportion to the number of squares in the wire segment under measurement. Note that the isolation resistances measured above for *s* > 2 *μ*m are greater than 10 GΩ [see Fig. 3(c)]; therefore, our measurements of multiple isolated implant lines make us very confident that the reduced resistance between these contacts here is due to the existence of the delta layer. We can therefore say with high confidence that we contacted the delta layer. The deviation from zero volts at zero current in the four terminal measurement is believed to be due to an offset in the differential amplifier used for the measurement. Looking more carefully at the results and using the STM image of the completed pattern (not shown), we estimate the wire under measurement in the 4 pt case has (14.7 ± 2) squares, corresponding to an ${R}_{\square }=(18.9\pm 2)\,k{\rm{\Omega }}$ for the wire. While this value is somewhat high compared to other work in the field^5^, this value is similar to other STM patterned devices measured using standard e-beam lithography based contacting method in our group, consistent with our assessment of good implant to dopant contact. Applying this ${R}_{\square }$ back to the 2 pt result, where we estimate (29.6 ± 3) squares total squares, the expected full wire resistance (without contacts) would be (560 ± 90) kΩ. The difference from the 2 pt measurement of ≈ 565 kΩ and the previous estimate can then be attributed to the nanowire to implant contact resistance, estimated to be (3 ± 45) kΩ each. While this value has a large uncertainty, it is a qualitatively small number that is certainly acceptable. The uncertainties reported for resistances are the standard deviations derived by propagating uncertainties and are dominated by the uncertainty in estimating number of squares contributing to each configuration. Comparing the 560 kΩ wire measurement, the > 10 GΩ implant isolation measurements and the geometrical considerations of source and drain, we conclude that we have made good electrical contact and measured an STM patterned device with a somewhat high ${R}_{\square }$.

## Discussion

As a consequence of the results presented, preimplant based electrical contacting of STM written nanodevices is not only possible, but feasible. At the outset of this work we identified a tension between close packing of the electrical lines to facilitate access by the STM and the requirement that these lines remain independent and isolated after thermal processing. As shown, the *s* = 2 *μ*m gap satisfies the requirements we set for all except the most extreme thermal process tested, which is much greater than what is used in actual practice. We did not explore any gap spacings between 1 *μ*m and 2 *μ*m, or other linewidths, so it may be that a further reduction in spacing or width could be realized, shrinking the excursion needed by the STM further or increasing the number of electrical lines possible. Additionally, the use of a heavier dopant like As could also enable tighter spacing and narrower lines, increasing the line density. Another approach can be to reduce the thermal budget required to prepare the surface for STM lithography, for example by using a silicon atom beam reduction method^14^.

Contact resistance between the STM written nanodevice and the preimplant wires reported here was based on the measurements performed at 4 K; therefore, further assessments of the contact resistance (with lower uncertainty) and yield of these contacts at temperatures below 4 K will be the subject of future studies. We also acknowledge that the preimplant templates have only been evaluated for electrical continuity and that measurements (e.g., coherence properties) sensitive to a dilute surface concentration of P atoms may be hindered if such a dilute density is present. However, to our knowledge, little is currently known as to whether the phosphine gas dosing strategy for forming nanodevices is immune to issues of dilute P doping throughout the surface and overgrowth, again, at densities well below the metal-insulator transition. Further, we note that the quality of the silicon surface is poorer above the implants, but the surface quality in the center regions of the circle defined by the implants is high quality (with dimer rows clearly resolved) and does not inhibit nanometer scale STM patterning. Finally, the implanted ion concentration for an unprocessed sample at the surface is estimated to be ≥ 5 × 10^19^ atoms/cm^3^, i.e., more than an order of magnitude greater than the density corresponding to the metal-insulator transition. After moderate thermal processing, e.g., ${D}_{{\rm{\Sigma }}}^{\ast } < 10$, the P ion density at the surface will increase due to diffusion and decrease due to sublimation. We believe that considerable margin exists before substantial depletion would harm the fidelity of the contacts considering that a good electrical connection is likely as long as the concentration is above the metal-insulator transition. But despite our success making contact to an STM written nano-device using ${D}_{{\rm{\Sigma }}}^{\ast }\mathrm{=9.5}$, minimizing the total amount of thermal processing is likely to maintain a high density of P at the surface and improve contact reliability and integrity. In the case of overly aggressive thermal processing, a silicon layer depleted of P may result that inhibits good electrical contact from the STM patterned device and the implanted contacts. Finally, based on the analysis on isolation resistance between implant lines with spacings > 2 *μ*m [see Fig. 3(c)], the statistical likelihood that we are measuring something other than the STM patterned nanowire is extremely small. Therefore, the measurements shown in Fig. 4 demonstrate successful implementation of this method in realizing electrical connections to a STM patterned nanodevice.

In summary, we have successfully demonstrated the feasibility of ion implanted degenerately doped wires in Si as an efficient and less complicated method for making electrical connections to nanoscale electrical devices. This approach completely eliminates the use of highly specialized tools, e.g., electron beam lithography, which enables a big technological advantage for many research groups for realizing electrical connections to nanoscale devices. The benefits of doing photolithographically defined ion implantation at the wafer scale dramatically reduces the overhead for fabrication and measurement of STM defined nanodevices compared to chip-by-chip electron beam lithography. Additionally, this approach connects the STM patterned region with the external electrical connections in-plane, increasing the number of available conduction channels between the two. This method also enables the ability to realize electrical connections to the STM patterned nanodevices *in situ*, and is a unique advantage of this method over contemporary contacting methods.The design rules and specifications demonstrated here provide room for a wide range of creative implementations, enabling a simpler path to challenging nanodevice and fabrication and measurements.

## Methods

We separate the methods into fabrication (clean room processing), UHV processing and electrical measurements.

### Device fabrication

Devices reported here were fabricated on Si substrates that are B doped with a resistivity of *ρ* ≈ 10 Ω ⋅ c*m* to 20 Ω ⋅ c*m*. Pre-UHV processing device fabrication was carried out at the wafer scale. First the alignment marks are defined on Si substrate using photolithography and subsequent “deep” etching of Si. Then the substrates are subjected to a standard RCA cleaning procedure^15^ to remove organic and metal contaminants followed by photolithography to define the regions that are ion implanted. Patterned substrates are then sent for external commercial ion implantation, specifically, a dose of 5 × 10^15^ atoms/cm^2^ phosphorus (^31^P) ions were implanted at an ion energy of 30 keV. According to the stopping and range of ions in matter (SRIM)^16^ simulations, the peak concentration of implanted ions is approximately 1 × 10^21^ atoms/cm^3^ at a mean depth of approximately 50 nm from the surface. To prepare samples for UHV processing, photoresist is spun on the substrates, which are then diced into 4 mm × 10 mm chips used in our UHV system. After dicing, the samples are RCA cleaned and loaded into UHV environment. (See UHV processing below.) Upon completion of the UHV processing, Ohmic metal contacts are fabricated by sputter deposition of Al, followed by photolithograhy to define an etch mask where the photo-mask is optically aligned to the etched alignment marks on the substrate [see Fig. 1(d). After etching the aluminum and removing the etch mask, the devices are annealed at 350 °C for 30 min in a N_2_ atmosphere to form an ohmic contact between the aluminum and P ion implanted contact pads^17,18^.

For the purpose of this study we repeated the standard protocol for several times in order to vary the total thermal activation of implanted ions. We use a etch process to define Ohmic metal contact pads. First, the sample is loaded to a sputter deposition system for metal deposition after a brief dip in 100:1 BOE in order to remove the surface oxide layer. In this study we used Al as our contact metal. Second, using photolithography the metal layer is patterned to define Ohmic contact pads and etched. Third, the sample is cleaned and annealed at 425 °C for 30 min in a N_2_ atmosphere in order to anneal the contacts.

### UHV processing

UHV processing of the samples was conducted in a dedicated system with a base pressure of < 7 × 10^−9^ Pa (< 5 × 10^−11^ Torr). Immediately after RCA cleaning, samples are loaded into UHV, followed by degassing > 8 h. Then each sample is flashed to ≈1200 °C according to the protocol [e.g., see Fig. 3(a)] described in the main text. The ultimate goal is to keep the sample at 1200 °C for 10 s to remove any oxide and prepare a 2 × 1 reconstructed surface while keeping the system pressure below 1.3 × 10^−7^ Pa (1 × 10^−9^ Torr). This is typically accomplished by flashes (brief, rapid heating) to lower temperatures, e.g., 1000 °C or 1100 °C for short times (8 s), where the decision to increase hold time or temperature for the next flash is based on maximum pressure of the prior flash. Once the 10 s mark is reached at 1200 °C, the temperature is quickly reduced down to 800 °C and then slowly ramped from 800 °C to the final temperature, e.g. room temperature, at a rate of 1 °C/s. The vacuum flashing procedures generally result in ${D}_{{\rm{\Sigma }}}^{\ast }$ values greater than standard post implant anneals for healing ion implant damage and no separate anneal is done to heal implant damage.

### Electrical measurements

Electrical measurements were carried out on a stage cooled by a closed-cycle cryocooler at < 4 K. Leakage resistance for the measurement system is approximately < 10 GΩ. For measuring isolation on the gap devices, a semiconductor parameter analyzer capable of simultaneously applying a voltage and measuring current was used. For the transport measurements of the STM written nanowire, a programmable constant-current source was stepped through an array of current values while a differential instrumentation amplifier was used to remove common mode voltage and amplify the signal measured by a voltmeter. Uncertainties of the measurements and the calculated quantities are smaller than the symbols in the plots; therefore, the uncertainties are not shown in the plots.
